# Legal aspects and government policy in increasing the role of MSMEs in the Halal ecosystem

**DOI:** 10.12688/f1000research.148322.4

**Published:** 2025-03-27

**Authors:** Yusup Hidayat, Aris Machmud, Sonny Zulhuda, Suartini Suartini

**Affiliations:** 1 Faculty of Law, University Al-Azhar Indonesia, Jakarta Selatan, Jakarta, 12110, Indonesia; 2 Ahmed Ibrahim Kuliyah of Law, International Islamic University Malaysia, Kuala Lumpur, Malaysia

**Keywords:** Business Ecosystem, Halal Product, MSMEs, Quadruple Helix

## Abstract

**Background:**

Indonesia currently could not make it to the top ten global halal due to some external and internal factors, although it is the largest Muslim country. In the external sector, the competition map is getting tougher, while internally, there are handicaps in the form of weak public awareness of competition. As a country with the largest Muslim population in the world, Indonesia can become one of the largest markets in the world. As indicated in the Global Islamic Economic Report (GIER), Indonesia is one of the top ten countries that control the Islamic economic market, yet it fails to dominate its Islamic market. Accordingly, the government needs to play a stronger role through regulatory policies to encourage the development of the Islamic economic ecosystem in Indonesia (halal value chain). The purpose of this study is to find out and analyse the existence of Micro, small, and medium enterprises (MSMEs) in Indonesia in the world’s market for Halal products and Services.

**Methods:**

The researchers have conducted a form of normative juridical research with the analytical descriptive method, namely by collecting, describing, analysing, and presenting what is ( *das sein*) and what ought to be ( *das sollen*).

**Results:**

Support from the government and stakeholders has not been optimal in improving welfare - capital access, management, and halal certification, so the existence of the global halal ecosystem has not yet impacted MSMEs’ ability to compete competitively.

**Conclusions:**

The conclusion of this study shows that the quadruple helix innovation system is capable of guaranteeing the development of MSMEs in a manner to achieves a high level of competitiveness - efficiency, and effectiveness of the products and services produced.

## Introduction

Indonesia can potentially be a leader in the global halal industry due to the supporting factor of the largest Muslim population. However, becoming a global leader in the halal industry will be lost if there is no other vital support from stakeholders, both in the form of policies and regulations related to halal certification, consumer awareness, research from academics and industry players, and community groups. This research shows why Indonesia should be the world’s biggest halal product market.

Indonesia currently could not make it to the top ten global halal due to some external and internal factors, although it is the largest Muslim country. In the external sector, the competition map is getting tougher, while internally, there are handicaps in the form of weak public awareness of competition, such as; Indonesians’ lack awareness of halal standardization, and an inadequate understanding of the law. However, apart from halal products - food, cosmetics, and medicines - Indonesia has begun to stand out in the fields of Islamic finance, halal tourism, and Muslim fashion in the world ( Permana, 2019).

Indonesia has enormous unused potential in the ecosystem of halal products and services in the world by involving all stakeholders to synergize with each other to increase the capacity and capability of Micro, small, and medium enterprises (MSMEs) to be competitive with imported products. MSMEs are only a bearing for development, the existence of MSMEs is still a complement to economic and legal politics in sustainable economic development. Despite the influence of economic globalization, the state’s commitment to the attainment of constitutional goals remains questionable ( Azis, 2018).

MSMEs are the foundation of the national economy in developing countries, where their contribution is up to sixty percent of the national income (GDP). Meanwhile, large industries only contribute forty percent to the GDP ( Suparji, 2020).

The global competitiveness of MSMEs and their contribution to national economic growth could be developed even further. They have been contributing for more than sixty percent of the GDP, absorbing up to ninety-seven percent of the workforce and fourteen percent of total national exports. This is due to the number of MSMEs that have reached more than fifty-seven million businesses, hence there is a need to enhance their capacity and capability. Switching the business ecosystem to a green economy poses a particular challenge for MSMEs in their effort to remain competitive. Therefore, the constraints they are facing need to be addressed immediately, enabling them to continue growing and become a mainstay in the national economy. Access to financing, lack of credit guarantees, production efficiency, and effectiveness remain serious obstacles for MSMEs to compete on a global level. Thus, solutions are needed to be sought through government regulation and policies ( Suparji, 2020).

Empowering MSMEs to have global competitiveness, the government issued regulations related to guaranteeing gratuitous halal products, besides that the government applies low taxation norms where the tax rate subsidy is zero point five percent for turnover up to 4.8 billion rupiah, as well as a loan interest subsidy of five percent and provides support for the digitization of the MSME market, as well as increasing business cooperation between large companies and MSMEs on a reciprocal basis that is accountable for realizing an integrated halal product supply chain ( Sauri, 2024). To improve the competitiveness of MSMEs in addition to those mentioned above, the government assists in adequate training and promotion ( Hidayat et al., 2025).

Such a condition is bound to harm the business world in general, especially if MSMEs’ existence is only considered as a complement to economic development. There is bound to be an increase in unemployment if the MSMEs’ competitiveness remains weak, which will make them act as mere spectators of the large potential of the global halal ecosystem ( Suparji, 2021).

For this reason, the government needs to support the existence of this largest business sector, namely MSMEs, by supporting the creation of a conducive ecosystem. Such *a halal*-based economy should be materialized through a policy of preference for domestic products because Indonesia is a country that has the largest Muslim market share in the world with more than two hundred million people ( Asep Firmansyah, 2022).

Limited capital is a common-place problem in MSMEs. It is evident that financial literacy, both sharia and conventional, has only reached seventy-six percent. This means, the potential to transform and digitize finance for MSMEs in increasing their production capacity as well as to improve the quality of their products. One of the difficulties of MSME actors in obtaining capital for MSME development is that capital owners do not yet have a prominent level of confidence in lending to MSME business actors ( Asep Firmansyah, 2022).

They still lack confidence in the success of MSME businesses. They also have to increase production capacity and quality, in addition to their managerial capabilities. It is specifically related to financial administration to facilitate financial inclusion and the use of information technology in increasing business opportunities and expanding market share ( Asep Firmansyah, 2022).

The contribution of MSME business units to Indonesia’s economic growth has been highly significant as they contribute almost ninety-nine percent of all business units. Therefore, there is a high chance that MSMEs will become major players in Indonesia’s and the world’s halal product ecosystem. Accordingly, the government needs to empower MSMEs by collaborating in expanding market access through various digital marketplace platforms and digitizing their finances ( Hartomo, 2020).

The *halal* ecosystem has a huge potential that can be developed by MSME businesses. There is a high demand for halal products in the global level and the world Muslim market share offers a very lucrative potential for business actors including MSMEs - both in developing and developed countries. For such a reason, a new orientation is needed in shaping the world halal market through the transformation of the halal industry ( Benuf & Azhar, 2020; Ali, 2021).

The progress of MSMEs has not been maximized due to limited access to capital and credit guarantees as well as the budget for obtaining halal product certificates ( Nugraheni, 2020).

The weakness of Suparji’s research is that it tends to place greater emphasis on legal reform that is adaptive to all changes in the business environment to support green economic ecosystems and MSME business development through government facilities and policies. Globalization and free markets bring intense competition, whereby only products with competitive advantages can survive in the business world, including MSMEs. The weakness of Asep Firmansyah’s research states that MSMEs prioritize domestic market share. In Hartono’s research, MSMEs can become major players in the world’s halal ecosystem through collaboration and utilization of digital media in the financial system and market access. Article Benuf & Azhar and Ali said that MSMEs can develop by transformation of the halal product ecosystem and a new orientation in the formation of the halal industrial market. Nugraheni’s research emphasizes that MSMEs are still inferior due to a lack of access to financing and halal certification.

Based on the state of the art above, the author states that there are differences in the object of research and the scope of research related to MSMEs. The novelty of this study is to increase the role of MSMEs in supporting Indonesia to be a leader and control the market share of the global halal products and services industry. The question is; how to make Indonesia’s MSMEs soar high in the world’s market for Halal products and Services?

## Methods

The Authors have conducted a form of normative juridical research with an analytical approach based on statutory rules to solve problems that exist within positive law also referred to as doctrinal legal research. In normative law methodology, this can be done through the search for principles, rules, and legal systematics, both codified as well as in the form of legislation ( Benuf & Azhar, 2020; Ali, 2021). Experts say that normative juridical research is a legal study that originates from positive or dogmatic legal norms - where the law as a system of norms and rules originates from legal products, both from regulators and court decisions as well as opinions of other experts to produce arguments, theories or a new concept as a prescription in solving the problem under investigation ( Ali, 2021).

Bambang Waluyo argues that normative juridical research is a document study using legal sources in the form of regulations and decrees and also using library sources to analyse and resolve legal issues. This study aims to get an objective description of a situation in a complete and detailed manner regarding a collection of arrangements regarding Islamic finance and MSMEs ( Kholid, 2018).

This form of research is intended to be a statute approach research, namely research that aims to track and analyze based on material in laws and regulations and analyze between *das sollen* and *das sein* -between what ought to have happened and what has happened ( Efendi and Ibrahim, 2016).

The analytical method used in this research is prescriptive analysis. Collection of legal materials in the form of literature studies through journal, literature books, statutory regulations such as the Monopolistic Practices and Unfair Business Competition law, Sharia Financial, Halal products are the responsibility of the government through the Halal Product Guarantee Agency (BPJPH) has functions based on presidential regulations, as well as data collection, through electronic media related to the problem ( Hafidz et al., 2018).

The criteria for financial inclusion is the community’s ability to access the formal financial system. The low level of financial inclusion is because there are barriers and access to formal finance (loan sharks). Inclusive finance can answer these reasons by providing many benefits for the community, regulators, government, and the private sector. Financial exclusion is the level of society that access the formal financial system at low and sustainable costs. Communities classified as having low financial inclusion are people with disabilities, low-income communities, and communities in disadvantaged, frontier, and outermost areas. Apart from that, workers without legal identity and small companies are un-bankable. Therefore, government policy should adopt the nine principles stated in the 2010 Toronto Summit on Innovative Financial Inclusion – leadership, diversity, innovation, protection, empowerment, cooperation, knowledge, proportionality, and frameworks ( BI, 2018).

This research was conducted in 2023 and has been presented in international seminars, strategies for finding legal sources from various sources, either through published or published websites, and relevant journals in 2018-2023. The analytical method used is a descriptive and prescriptive analysis based on sources and secondary laws that are collected, sorted, and interpreted to answer the problem formulation ( Indrawan & Nahartyo, 2020).

## Results

### MSMEs in the world’s market halal product

Indonesia is currently still the largest consumer in the world in the world’s ecosystem of halal products and services. Indonesia has the potential to be on top based on indicators and instruments that support this - large potential consumers of halal products and services, availability of certification bodies, and recognized fatwa institutions ( Syamsu, 2023).

In addition to the foregoing, product quality assurance is supported by regulations ( President Republic of Indonesia Law of the Republic of Indonesia Number 33 Year 2014 on Halal Product Assurance, 2014), policies, and also qualified halal auditors so that Indonesia can play a more active role in the global halal export market. Indonesia’s weakness is undeniable that the limited non-production budget for the processing of halal certification can potentially lead to a high-cost economy. This plays a part in reducing the level of competitiveness ( Syamsu, 2023).

Orosz in Suparji states that MSMEs can make a more optimal contribution by increasing access to capital and assistance to increase effectiveness and efficiency in production so that they have a level of global competitiveness in line with the conditions faced as a consequence of the free market ( Suparji, 2021).

The obstacles that are often faced by MSMEs are the lack of financial inclusion and literacy; the protection of MSMEs requires a different approach vis-à-vis the existing regulations. Thus, there is a need for an adaptive legal reform granting them equal treatment with large and established businesses. The existing fiscal and monetary policies do not support the existence of MSMEs - there are no subsidies and the implementation of a financing system (equated with large businesses) ( Azis, 2018).

Based on the foregoing it can be concluded that the achievement of the purposes of the 1945 Constitution through the development of people’s businesses and MSMEs has experienced ups and downs since the era of independence, as a result of which there are still groups of people who are classified as underprivileged ( Azis, 2018).

Facilities need to be provided by the state specifically to MSMEs - without compromising the essence of maintaining halal integrity. The provision of a simple halal product guarantees registration form and the imposition of a processing fee adjusted to the business conditions and sales turnover of each of these MSMEs so that through halal certificates and other documents they can expand their market access – going from local to global ( Ahyar, 2020).

Partnerships between large entrepreneurs and MSMEs can create prosperity for stakeholders and they can also contribute to the national economic growth through the fulfillment of commitments and business ethics. However, an unequal bargaining position frequently occurs as a result of setting detrimental partnership agreement clauses ( Tanjung, 2022).

For such reasons, the role of the government and corporate companies - through CSR - is needed to provide protection and progress for MSMEs. It needs to be based on sociocultural aspects and mutually beneficial partnerships among stakeholders - whereby MSME products are absorbed by corporations making it easier to market them ( Kelly et al., 2020).

Partnership agreements need to be simple and understandable to both parties and applicable in complex circumstances. Policies in business competition in Indonesia are aimed at encouraging the equal distribution of business opportunities, legal certainty, and ease of access to markets, capital, and technology for MSMEs following the mandate of the Monopolistic Practices and Unfair Business Competition law ( Kelly et al., 2020).

The halal ecosystem is supported by the seriousness of the government through regulations of halal products. Halal products are the responsibility of the government through the Halal Product Guarantee Agency (BPJPH) which has functions based on presidential regulations. said body has the following authorities ( President Republic of Indonesia Law of the Republic of Indonesia Number 33 Year 2014 on Halal Product Assurance, 2014) see ( Government Regulation in Lieu of Law No. 2 Year 2022 (“Perppu No. 2”), 2022):

a. Formulating and establishing policies related to the halal nature of a product;
b. Establish SOPs, norms, and criteria related to the halal nature of a product;
c. Issue halal certificates that have received halal fatwas from MUI (Indonesian *Ulemas*’ Council) and revoke certificates for products from within and outside the country;
d. Registering halal product certificates from within and outside the country.

The determination of halal products begins with a review and research process by LPPOM (Institute for the Assessment of Cosmetic Drugs and Food) by considering the halal nature of a product, both goods and services, from consumer goods to kitchen utensils and clothing - all under the Islamic law. Business actors who have obtained Halal certificates are required to declare and disclose both on product packaging which is easily visible and difficult to be damaged or removed.

However, Indonesia has the potential to become a world market for halal goods and services. Its failure as the largest Muslim country in the world needs attention from stakeholders. This research shows why Indonesia should be the world’s biggest halal product market. Indonesia currently could not make it to the top ten global halal due to some external and internal factors, although it is the largest Muslim country.

In the external sector, the competition map is getting tougher, while internally, there are handicaps in the form of weak public awareness of competition, such as; Indonesians’ lack awareness of halal standardization, and an inadequate understanding of the law. However, apart from halal products - food, cosmetics, and medicines - Indonesia has begun to stand out in the fields of Islamic finance, halal tourism, and Muslim fashion in the world ( Permana, 2019).

Indonesia has enormous unused potential in the ecosystem of halal products and services in the world by involving all stakeholders to synergize with each other to increase the capacity and capability of MSMEs to be competitive with imported products.

Partnerships between large entrepreneurs and MSMEs can create prosperity for stakeholders and they can also contribute to the national economic growth through fulfilment of commitments and business ethics; however, an unequal bargaining position frequently occurs as a result of setting detrimental partnership agreement clauses ( Tanjung, 2022).

For such reason, the role of the government and corporate companies - through CSR - is needed to provide protection and progress for MSMEs ^.^ It needs to be based on socio-cultural aspects and mutually beneficial partnerships among stakeholders - whereby MSME products are absorbed by corporations making it easier to market them ( Mantili, 2020).

Partnership agreements need to be simple and understandable to both parties and applicable in complex circumstances. Policies in business competition in Indonesia are aimed at encouraging the equal distribution of business opportunities, legal certainty, and ease of access to markets, capital, and technology for MSMEs by the mandate of the Monopolistic Practices and Unfair Business Competition law ( Mantili, 2020).

An agreement is binding on the parties who make it, all clauses are agreed upon by the parties and the parties are responsible for enforcing the partnership agreement ( Kelly et al., 2020).

There are many companies in the market, both large businesses and SMEs that allow for economies of scale to occur through partnerships with one another. However, these partnerships do not guarantee a business ecosystem that is free from competition between them because of the subjective desire of the parties to create and abuse market power - cartel or other illegal behaviour that has the potential for criminal prosecution. For such reason, the state can intervene to prevent unhealthy business actions from occurring, especially for MSMEs which constitute the largest number of business units that can absorb labour.

Therefore, the capacity and capabilities of MSMEs need to be protected to enable them to be competitive. One form of capacity building is forming partnerships with large business actors. However, such partnerships are frequently more profitable for large businesses due to their greater bargaining power, which ultimately results in unfair business competition through unreasonable unilateral pricing. Such abuse of bargaining power can potentially lead to reduced competition due to obstacles in the ease of doing business and fair business, it is at this point that the state through its policies and regulations provides legal protection ( Stiglitz, 2021).

Sudaryanto and Wijayanti stated that MSMEs need internationalization to be able to explore market access opportunities abroad through partnerships so that they can be competitive amid globalization and high competition, and thus have the ability to face global challenges. Such internalization takes the form of increasing product and service innovation, developing human resources and technology, and expanding marketing areas ( Hadi & Setyawati, 2020).

It needs to be noted that partnerships bring legal consequences to the parties agreeing. Agus Budiarto and Ridwan Khairandy state that companies are legal subjects, including companies that are bound by civil law by the provisions of Book III of the Indonesian Civil Code ( Prabowo, M Shidqon and Umami, 2018).

Corones in Michael Schaper suggests that competition policy is needed to ensure the creation of a perfect competition market without any restriction and for this reason competition law is needed to ensure that business actors carry out business in good faith and refrain from unhealthy business conduct. At the same time, the competition law is in the form of regulations that are related to certain forms of competition.

However, it also includes other measures including limiting the role of the state in market intervention, unfair trade practices - a ban on anti-competitive agreements; prohibition of abuse of dominant market position; prohibition of anti-competitive mergers and acquisitions - as it would distort perfect market competition ( Michael Schaper, 2021).

MSMEs are entities that are vulnerable to being affected by behaviour that deviates from fair trade - abuse of bargaining power, cartels, and abuse of dominance - as happened in the Philippines and Fiji - through exclusive transaction arrangements. ASEAN has agreed to apply general competition laws similar to the prohibition of anti-competitive agreements (including cartels), abuse of dominant position, and anti-competitive mergers and acquisitions ( Michael Schaper, 2021):

a) Cartels and other anti-competitive agreements. Cartel is considered the most serious violation of competition law and it generally carries the most severe penalties (fine and, in some jurisdictions, possibly imprisonment). A cartel is an agreement in which competitors in the same market agree to fix prices, share markets, bid rigs, or limit production or supply. In general, other anti-competitive agreements that have the effect of distorting competition in the market (unbalancing the playing field) are also prohibited.
b) Abuse of dominant position. Businesses that hold dominant market positions must not exploit their positions to the detriment of their customers or exclude competitors from the market. All ASEAN competition laws contain prohibitions of this type. Proving these types of violations can be very difficult for competition agencies, especially those with less experience.
c) Anti-competitive amalgamation. Mergers that result in significantly reduced competition in the market after the merger takes effect, may be prohibited by the competition authorities. The policy aims to ensure that competition remains in the market after the merger so that consumers still have a choice.

Indonesia is currently still the largest consumer in the world in the world’s ecosystem of halal products and services. Indonesia has the potential to be a winner based on indicators and instruments that support this - large potential consumers of halal products and services, availability of certification bodies, and recognized fatwa institutions. In addition to the foregoing, product quality assurance is supported by regulations ( President Republic of Indonesia Law of the Republic of Indonesia Number 33 Year 2014 on Halal Product Assurance, 2014), policies and also qualified halal auditors so that Indonesia can play a more active role in the global halal export market. Indonesia’s weakness is undeniable in that the limited non-production budget for the processing of halal certification can potentially lead to a high-cost economy thereby reducing the level of competitiveness ( Syamsu, 2023).

### Quadruple helix system

Cummings, Cross & Cummings, Davenport & Prusak state that development must refer to Sustainable Development (SDGs) adopted at the 2015 UN General Assembly session (United Nations, 2015). In this context, the collaboration of stakeholders with community members plays a highly significant role in finding innovative solutions through knowledge sharing to achieve effective use of resources through social networks and related local communities ( Hakeem et al., 2023).

Economic-based knowledge has become available as a result of globalization in sustainable development through innovation and knowledge transfer as well as collaboration among all stakeholders (Triple Helix (TH) Model). However, Milner et. al, Kimatu believes that TH is still ineffective due to the lack of support from civil society for it to become the Quadruple Helix (QH) model as the fourth pillar towards achieving sustainable development. There is an urgent need for civil society involvement in such a helix model because it places greater focus on the micro perspective so that dynamic relationships, synergy, collaboration, coordinated environment, and value creation activities accelerate problem-solving due to the maximization of effective resource exploration ( Hakeem et al., 2023).

Likewise, global Halal products and services involving elements of government, entrepreneurs (investors), academics, and also the community (producers and consumers) form a circle involving mutually linked elements aimed at realizing Sustainable Development Goals, ensuring that people are free from hunger, stunting, poverty rates are reduced, especially in countries with Muslim populations and globally ( Krisna et al., 2023; Sukoso et al., 2020).

The government is the foundation for all policies and becomes a political power in the discovery of these policies and regulations. (Yolanda, 2021) Multi-stakeholder collaboration in the quadruple Helix element can potentially enhance the development of MSMEs with more added value ( Windhyastiti et al., 2022).

**Figure 1.**
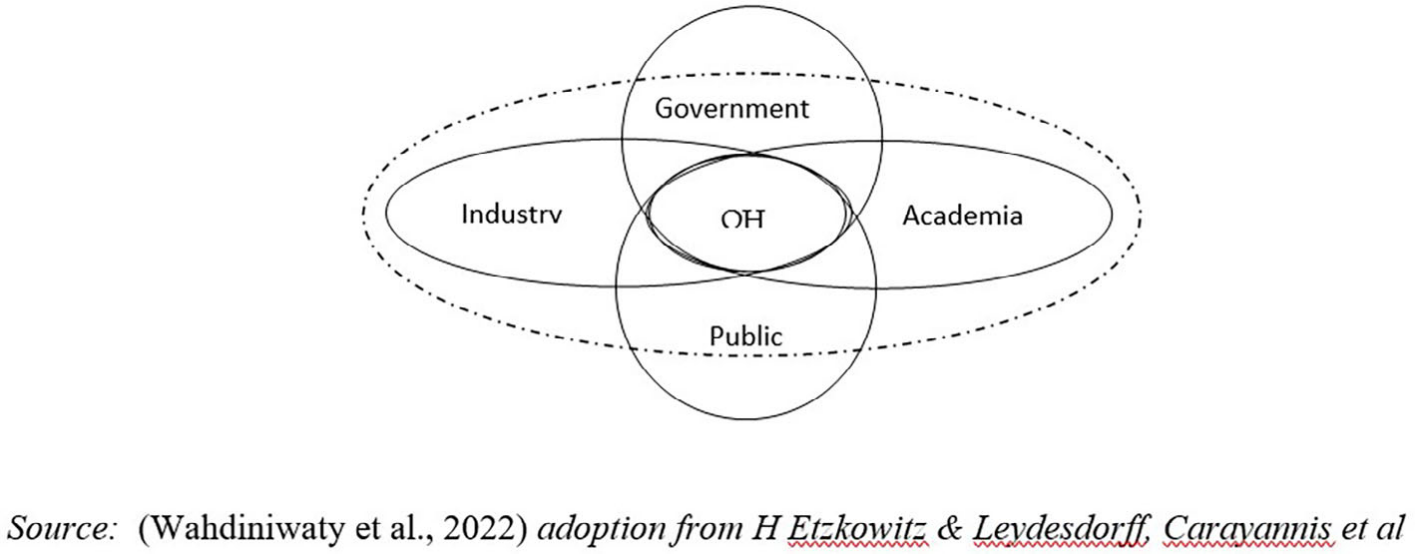
Five Institutional Model of Quintuple Helix Innovation (QHI). Source: This figure/table has been reproduced with permission from Wahdiniwaty et al. (2022).

H Etzkowitz & Leydesdorff, Carayannis *et al* state that in the system model an environmental ecosystem is needed. Therefore, the effectiveness of this quality system achieves sustainable development due to embedding social environmental factors in QHI is not only an aspect that becomes an ecological area for QH actors to work together and interact with each other but more than that the environment can influence the formation of innovation in a sustainable manner in the QHI model, it can be seen that the social environment represents, frames, and simultaneously influences the cooperation and interaction of the five helices ( Figure 1) ( Wahdiniwaty et al., 2022).

Environmental factors are disruptive as innate and global external influences so they require adaptive behavior and social interaction from the helix to create balance and harmony ( Wahdiniwaty et al., 2022).

a. Government Roles

The government’s seriousness in enhancing the transformation and digitization of MSMEs was emphasized by President Joko Widodo who stated that through collaboration between stakeholders, it is possible to improve and accelerate the process of transformation and digitization in the Islamic financial sector to provide added value to MSMEs in Indonesia.

The lack of skilled personnel in the Islamic financial services sector is also due to cross cross-placement of labour, whereby the banking sector is mostly filled with non-Islamic finance graduates. The development of financial technology (Fintech) is yet to be utilized properly ( Sukoso et al., 2020).

Islamic financial digital literacy is an important indicator of success or failure regarding the understanding of Islamic financial products by the wider community. The lower the literacy index value, the lower the level of public understanding of Islamic financial literacy ( Fawaid & Utama, 2022).

The government can encourage the use of digitalization of Islamic finance through socialization to the public, both through online and offline services, as well as commercial advertisements both print and television, or by conducting FGDs with interested parties. Improved products and services, enhanced human resources, and a more competitive profit-sharing ratio are required to increase financing for MSMEs and can compete with conventional banking ( Fawaid & Utama, 2022).

One of the roles of the government in developing the halal industry is through strategic policies to utilize the potential of the halal industry, which supports the productivity of MSMEs in meeting international demand for halal products as well as encouraging the public to use domestic halal products. Regulatory support is no less important in supporting the development of the Indonesian halal industry. This is reflected in the seriousness of the Indonesian government in issuing regulations related to halal certification, namely Government Regulation (PP) Number 39 of 2021. The strategy adopted by the government is as follows ( Ahmed, 2023):

a. Halal Product Policy Development;
b. Infrastructure Expansion;
c. Halal Product Guarantee System;
d. Fiscal and Non-Fiscal Incentives – Monetary, Financing;
e. International Collaboration for Halal Ingredients;
f. Increasing Market Access;
g. National Halal Certification;
h. Mutual Recognition Agreement (MRA) with other countries;
i. Festival to Promote Literacy Initiatives.

The position of Indonesian MSMEs is comparable to those in India. In addition to access to formal finance, MSMEs in India have management boundaries and the complexity of MSME procedures due to the banking adequacy ratio based on Basel III ( Nikam, 2019).

Such challenges call for a government policy to overcome the challenges. Such policies included fiscal as well as non-fiscal policies. Indonesia has implemented fiscal policies for MSMEs based on Government Regulation (PP) No. 23 of 2018, applying a 0.5 percent tax for turnover of no more than IDR4.8 billion per year ( Kemenkeu, 2022).

In addition to that, the government can potentially offer convenience through non-financial policies, namely by facilitating the process of halal certification for MSMEs. Generally, MSMEs only have a budget for production – all of the foregoing to enable MSMEs to be more productive and competitive in the halal industrial ecosystem. Likewise, in India, the government also encourages the empowerment of start-ups and MSMEs through the benefit of fiscal zero percent for MSMEs ( Nikam, 2019).

b. Academy Roles

The role of the education sector is supported by the National Science and Technology Law. It is to make Indonesia a ‘knowledge economy’ that will drive economic growth through innovation and access to information. There is awareness that this requires collaboration among stakeholders - government, academia, the private sector, and civil society.

With the above strategy, Indonesia has the potential to increase spending by the Indonesian people on Indonesian halal goods and services by around fifteen percent of the total population with an estimated income of more than two hundred and eighty-one billion dollars in 2025 and make Indonesia the largest consumer in the world, by contributing eleven-point thirty-four percent of global halal spending ( Ahmed, 2023).

Kartasasmita states that the strong growth of the Sharia economy and the halal industry has resulted from several factors; a large Muslim population, growing awareness of Islamic values related to halal products, and a national strategy targeting the development of halal products and services. Bank Indonesia projects that the domestic halal value chain sectors which include agriculture, halal food and beverages, Muslim fashion, and Muslim-friendly tourism will grow by four point five-to-five-point three percent in 2023, contributing more than twenty-five percent to the national economy ( Ahmed, 2023).

c. Community Roles

Local halal businesses supported by their communities will be able to compete, absorb a lot of labour and contribute to a sustainable economy. Community empowerment through the creation of community-supported job opportunities will increase people’s income which in the long run will have an impact on national economic development and can potentially bring about stability in the community industries ( Ahmed, 2023).

The role of academics in developing halal products is through the creation of innovations that will be socialized and implemented by the community so that they can develop product diversification and halal product certification services. Networking and collaboration between the two pillars of the Quadruple Helix is the sharing of this knowledge that is essential to promote halal business effectively, educate people about the importance of halal compliance, and build connections with the Halal ecosystem, namely individuals, organizations, and industries ( Ahmed, 2023).

Increasing awareness of the halal business will encourage the development of the halal industry as a whole. Local halal businesses - in this case, MSMEs - can play an important role in supporting and promoting halal businesses and empowering their communities through the application of halal principles ( Ahmed, 2023).

d. Industries Roles

The halal industry sectors that can be developed and improved by business actors, both large industries as well as MSMEs, namely the food and beverage industry, the halal tourism industry, the beauty and cosmetics industry, the fashion industry and the financial and pharmaceutical services industry.

The government - Ministry of Cooperatives - is one of the important pillars in empowering MSMEs through policies that support the economic ecosystem of MSME communities through convergence with relevant institutions, including management and technical assistance in developing business sustainability so that it can increase motivation, increase the capacity of MSMEs so that it can improve performance and ease of access to capital ( Analia, 2020).

Most MSMEs (more than sixty-five million business units) are household businesses that have many limitations in both market access and capital - but behind these limitations, MSMEs can absorb more than one hundred and twenty-three million workers - so that assistance through government policies can encourage increased MSME performance as well as the country’s economic growth (contributing almost two-thirds of national GDP).

The role of the government in encouraging MSME businesses can be through tax facility policies and capital assistance through people’s business credit, both through bank and non-bank financial institutions - with low interest rates - so that these MSMEs can develop and be competitive. So, the helical role of this government element must focus more on empowering MSMEs in the financial aspect through integrated financing sourced from the APBN and developing business capacity and high competitiveness that is export-oriented ( Limanseto, 2023; Cirprandy Riopaldo Tambunan, 2023).

**Figure 2.**
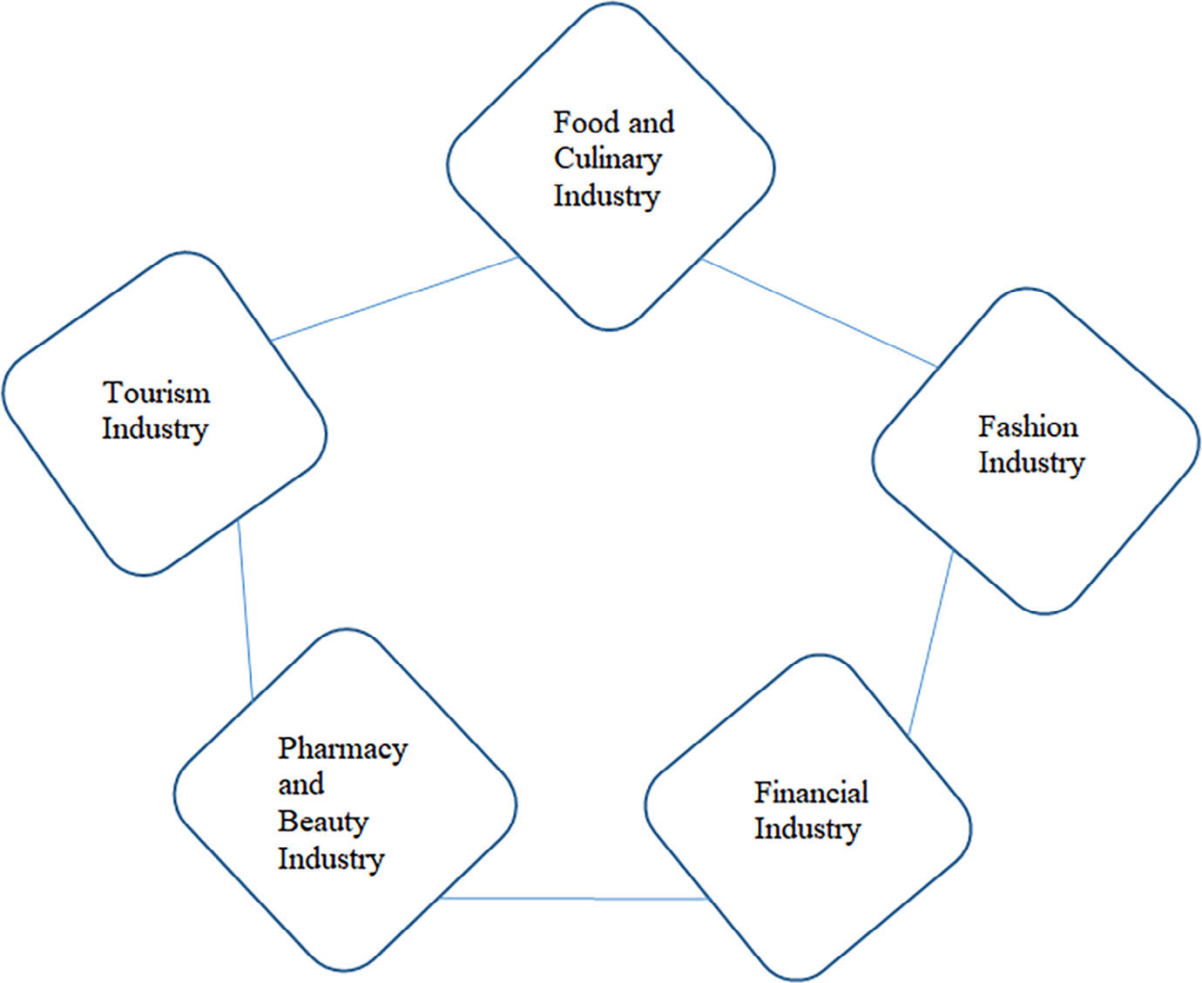
Halal ecosystem. Source: This figure/table has been reproduced with permission from Ahyar (2020).

The role of the Academic pillar in the development of MSMEs will always be sustainable in the tri-dharma tasks of higher education - teaching, research, and community service - which can increase the capacity and capabilities of MSMEs through the role of community services. Academics can contribute through socialization and training in accounting management, management and marketing as well as product development, to make it easier for MSMEs to get easy access to financing from banking financial institutions - because one of the weaknesses of MSMEs is un-bankable ( Tanjung, 2024). In addition, the role of academics can also establish networks between other helixes - society, government, and industry - through cooperation that mutually supports the development of MSMEs ( Humas, 2021). These figures indicate that the awareness and need for halal products in Indonesian society continues to increase – particularly for food and beverage, pharmaceutical, and cosmetic products - although it is still supported by imported products. It gives a signal that the time has come for the domestic halal industry to take this opportunity to become a host in the world of global halal products. Unfortunately, the government has also been rather serious about encouraging the growth of sharia business in Indonesia through regulation. In the halal product ecosystem, we can break down the sectors of goods and services that can be transacted in the Sharia business world both locally and globally, as shown in Figure 2 ( Ahyar, 2020).

The Global Islamic Economic Report for 2019-2020 reports that on a global scale, Indonesia is not included in the top 10 countries with the best halal food performance in the world. For such a reason, it is necessary to have enforcement from business actors and other stakeholders to ensure that we can become a centre for products and also a global Sharia economic market ( Sukoso et al., 2020).

Tobing and Adrian state that the transformation and digitization of Islamic finance facilitate the flow of trade transactions in the business world, with a reach beyond inter-jurisdictional boundaries. It is through the use of technology that transactions can be carried out in a free space and time thus the digitization of payments can improve the global economy. However, the role of transformation and digitalization must involve the quadruple helix - the government or public authorities, industry, educational institutions or institutions, and the community as users. The low level of Islamic financial literacy, namely only 8.93 percent of the national average, indicates that most Indonesian people do not understand the characteristics of formal financial institution products and services. This is evident from the level of public ownership of Islamic financial accounts and Islamic products which is still relatively very small. The level of Islamic financial literacy can be measured using four indicators, including the following ( Fawaid & Utama, 2022):

(1) Basic financial knowledge (prohibition of usury, *Gharar*, and prohibition of *maysir*).
(2) Loans/credit ( *mudharabah* financing, *musyarakah* financing, *murabahah* financing, *salam* financing, *istishna* financing, *ijarah* financing, and *qardh* financing).
(3) Investment/savings (types of investment and types of savings recommended in Islamic finance).
(4) Insurance, (sharia */takaful* insurance). With the development of the digital world, increasing Islamic financial literacy can be carried out through the transformation and digitization of Islamic finance.

Based on Financial Services Authority (OJK) data - SNLIK 2022 results - financial inclusion has increased significantly from 76.19 percent in 2019 to 85.10 in 2022 thus showing that the gap between literacy and inclusion levels has been decreasing, namely from 38.16 percent in 2022 to 35.42 percent. Based on the survey, data on the financial literacy index and also financial inclusion shows that as of 2022 financial literacy increased by thirty-eight-point zero-three percent compared to 2019, while the financial inclusion index in 2022 increased by thirty-eight-point sixteen percent from 2019, as shown in Table 1 ( Keuangan, 2022).

**Table 1.** Comparison between financial literacy and inclusion indexes in 2019 and 2022. Source: OJK (2022).

Index	2019	2022
Literacy	38.03%	49.68%
Inclusion	76.19%	85.10%
Gap	38.16%	35.42%

**Figure 3.**
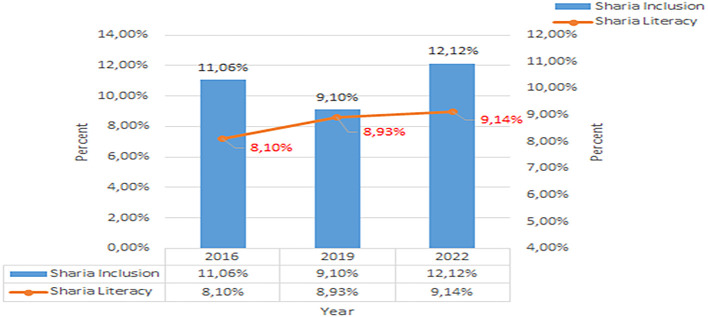
Data on Sharia financial literacy 2016-2022. Source: From Press release Indonesia FSA ( OJK, 2016, 2022a, 2022b).

Based on this data, in 2019, nationally it was concluded that Islamic financial literacy was still relatively low. From 2016 to 2019, there was only an increase of 0.83%, namely to 8.93%. Unlike the case with conventional financial literacy, from 2016 to 2019 there was an increase of 8.22%, namely to 37.72%. The development of Sharia financial inclusion and literacy per three years from 2016 to 2022 shows in Figure 3 below ( OJK, 2016, 2022a, 2022b).

**Figure 4.**
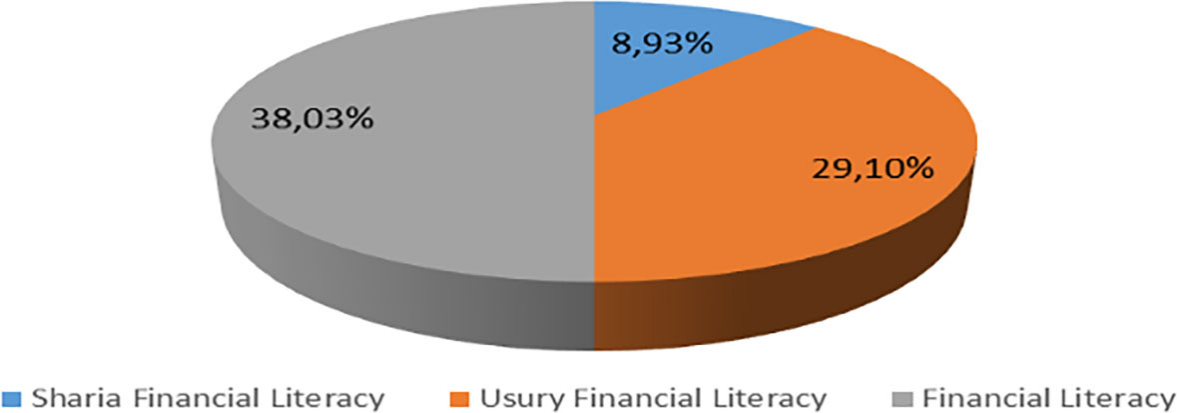
Data on Usury and Sharia financial literacy 2019. Source: From Press release Indonesia FSA ( OJK, 2022b).

**Figure 5.**
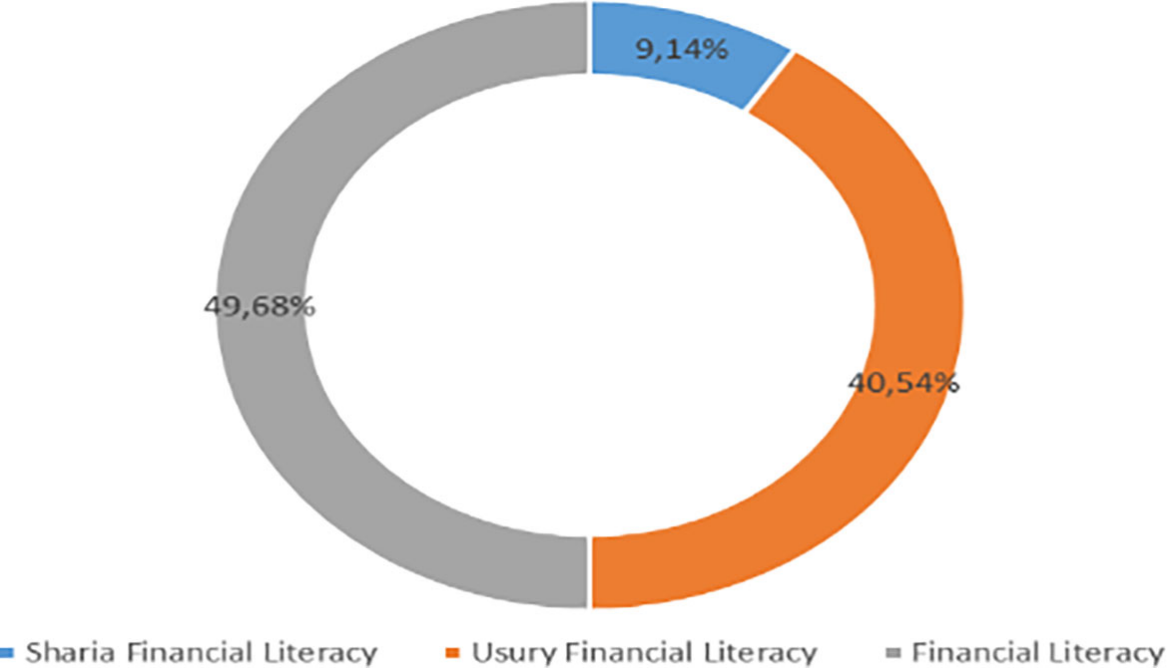
Data on Usury and Sharia financial literacy 2022. Source: From Press release Indonesia FSA ( OJK, 2022b).

Increasing Islamic financial literacy is fundamental for the development of Islamic finance. This makes it interesting to study more in-depth, hence the authors are interested in examining this in an analytical descriptive study. This is done to explain the various efforts and strategies carried out by the four elements, namely the government or public authorities, industry, educational institutions or institutions, and the community as users. Based on SNLIK 2022, it shows that the level of sharia financial literacy has increased from 8.93% in 2019 to 9.14%, while the level of financial inclusion has also increased from 9.10% in 2019 to 12.12% in 2022. Graphical representation of Financial Sharia and Usury Literacy of the year 2019 is shown in Figure 4 and Graphical representation of Financial literacy data in 2022 can be seen in Figure 5 below ( OJK, 2022b).

### Halal product, fashion, service ecosystem

Halal product ecosystems function through supply chains that absorb the legal approach of supply and demand as well as enablers or supports. Meanwhile, from the supply side, it is necessary to have an industrial sector that offers quality halal products and is needed by the community, namely halal goods and services. At the same time, from the demand side, there is a great number of consumers/people who need halal products, whereas on the supply side, the high interest in halal products encourages all countries, both Muslim and non-Muslim, to offer premium halal products to form a perfectly competitive market through the provision of infrastructure, supply distribution and support for research and development of halal products (for product competitiveness) – through product, service, and process innovation support ( Brier & Jayanti, 2020).

The halal industrial ecosystem requires a supply chain system to ensure the sustainability of quality and efficient halal products, namely products that are presented by the law of demand and supply, without a high-cost ( Brier & Jayanti, 2020).

The demographics of the world’s Muslim population in 2060 are estimated to reach the level of two point nine billion people. This offers a potential for increasing the world’s Islamic economy with a growth rate of consumption of halal products of five-point two percent per year or a value of two point two billion US dollars per year, which is a huge potential ( Brier & Jayanti, 2020).

It is a great challenge for Indonesia to become a global player in the world Sharia business. With the world’s largest Muslim community, it also has a productive population of nearly 68% of its total population; in 2019/2020 Indonesia ranked fifth in the world after Malaysia, UAE, Bahrain, and Saudi Arabia – previously ranked in the top ten ( Brier & Jayanti, 2020).

## Discussion

The message in the Qur’an Surah *Al Baqarah* Verse 168 requires that something *Halal, and Thoyib* is an order from Allah SWT as a form of self-serving to be free from sin as well as the personal right of Muslims that must be respected. As a guarantee of quality with zero tolerance for unclean materials or substances into *halal* materials, whether intentionally or not – contaminated with *haram* (forbidden) materials ( Syamsu, 2023).

*Halal* products contain spiritual and safety (quality) aspects that require them to be free from the main elements of the ingredients or the results of contamination of prohibited materials so that they are suitable for consumption by Muslim and non-Muslim consumers globally. The increasing trend of global *halal* products and services has been creating great potential in the national economy over the years, with an economic potential of nearly three trillion dollars ( Syamsu, 2023).

The potential for the *halal* industry is very promising because the ecosystem can become a prima donna in the world economic sector. The development of halal products and lifestyles is experiencing an increasing investor interest in building and developing the Sharia industrial sector in Indonesia as well as with Indonesia having the largest Muslim market share in the world as well as government support in encouraging the Indonesian halal industry to become a champion in the global halal industry ( Ahyar, 2020).

## Conclusions

The results of this study indicate that the quadruple helix innovation system can help to ensure that the legal system has strong binding power while providing a multiplier effect on the national economy. The quadruple helix quality system creates cohesiveness of the government through fiscal and non-fiscal incentives, as well as policies and regulations that support the productivity of MSMEs in halal goods and services. In the academic sector, with support from the National Science and Technology Law, which can be expected to encourage economic growth through innovation and access to information. Collaboration in the quadruple helix between government, academia, industry, and community makes it possible for MSMEs to develop and have global competitiveness in the Ecosystem of Halal Products and Services. The halal ecosystem requires a halal social environment that must be adaptive so that stakeholders can at all times be agile towards any changes that occur.

### Limitation and further research

This study examines the impact of regulations and also the involvement of parties in the development of MSMEs in competition for halal products, to increase their role. Indonesia is in control of market share in the product ecosystem of halal goods and services. Research is not immune from imperfections. Therefore, it is necessary to study other factors that hinder Indonesia’s position in controlling the world’s halal products. The use of a multidisciplinary or interdisciplinary approach can be considered in further research.

## Data Availability

No data are associated with this article.

## References

[ref1] Ahmed HM : How Can Gen Z Muslims Shake the Global Halal Industry. *The Halal Times.* 2023, April 12;1. Accessed May 18, 2023.

[ref2] Ahyar MK : Halal Industry and Islamic Banking: A Study of Halal Ecosystem Regulation in Indonesia. *Journal of Finance and Islamic Banking.* 2020;2(2):165–182. 10.22515/jfib.v2i2.1929

[ref38] Ali Z : Metode Penelitian Hukum. *Sinar Grafika.* Mataram University Press;2021. Reference Source

[ref40] Analia D : Peran Stakeholder dalam Meningkatkan Kinerja Usaha Mikro Kecil di Kota Padang, Sumatera Barat. *Industria: Jurnal Teknologi Dan Manajemen Agroindustri.* 2020;9(3):203–216. 10.21776/ub.industria.2020.009.03.5

[ref3] Asep Firmansyah RS : Ministry optimistic of halal ecosystem driving the national economy. *ANTARA News.* 2022, February 23. 1 Diakses 02 Maret 2023.

[ref4] Azis MH d M : *Pembangunan Ekonomi & Pemberdayaan Masyaakat Strategi Pembangunan Manusia dalam Perspektif Ekonomi Lokal* (Edisi Kedu). CV. Nur Lina dan Pustaka Taman Ilmu. 2018.

[ref5] Benuf K Azhar M : Metodologi Penelitian Hukum sebagai Instrumen Mengurai Permasalahan Hukum Kontemporer. *Gema Keadilan.* 2020;7(1):20–33. 10.14710/gk.2020.7504

[ref6] BI: Keuangan Inklusif. *Bank Indonesia Website.* 2018; pp.1. Accessed April 27, 2024. Reference Source

[ref7] Brier J Jayanti LD : *Strategi Nasional Pengembangan Industri Halal Indonesia.* Komite Nasional keuangan Syariah;2020; Vol.21(Issue1). Reference Source

[ref81] Efendi J Ibrahim J : *Metode Penelitian Hukum: Normatif dan Empiris (Pertama)* : Prenadamedia Group;2016.

[ref8] Fawaid MW Utama YY : Digital Literacy of Sharia Finance in Indonesia With a Quadruple Helix Approach. *International Conference on Islamic Studies.* 2022;3:318–325.

[ref26] Government Regulation in Lieu of Law No. 2 Year 2022 (“Perppu No. 2”).2022. Reference Source

[ref9] Hadi IRR Setyawati R : Partnership Agreement By Dominant Posission Undertakings: Legal Protection of Micro, Small, and Medium-Sized Enterprises. *PalArch’s Journal of Archaeology of Egypt/Egyptology.* 2020;17(3):1989–1996.

[ref10] Hafidz J Saragih YM Prasetyo T : Analisis Yuridis Kewenangan Komisi Pemberantasan Korupsi (KPK) sebagai Penuntut Pelaku Tindak Pidana Korupsi. *UNIFIKASI: Jurnal Ilmu Hukum.* 2018;5(1):33. 10.25134/unifikasi.v5i1.763

[ref11] Hafiz MA : Why Do We Need Local Halal Champions Everywhere. *The Halal Times.* 2023, April 14;1Accessed May 18, 2023.

[ref12] Hakeem MM Goi HC Frendy : Regional sustainable development using a Quadruple Helix approach in Japan. *Regional Studies, Regional Science.* 2023;10(1):119–138. 10.1080/21681376.2023.2171313

[ref13] Hartomo G : Terkuak, Ini Kontribusi UMKM bagi Perekonomian Indonesia. *Okezone. Com.* 2020, October 20. 1 Di akses 26 Februari 2021. Reference Source

[ref45] Hidayat AA Ikhsan F Hafid NS : The Future of MSMEs in Indonesia: Policy Directions for National Legal Formation in Supporting MSMEs Growth. *JUSTISI.* 2025;11(January):299–316. 10.33506/js.v11i1.3876

[ref43] Humas : Kembangkan Ekonomi Kreatif melalui UMKM. *Universitas Negeri Yogyakarta.* 2021, October 30; Accessed 8 January 2025. Reference Source

[ref42] Limanseto H : Dorong UMKM Naik Kelas dan Go Export, Pemerintah Siapkan Ekosistem Pembiayaan yang Terintegrasi - Kementerian Koordinator Bidang Perekonomian Republik Indonesia. *Siaran Pers Kementrian Koordinator Bidang Perekonomian Republik Indonesia.* 2023, August 24; Accessed 8 January 2025. Reference Source

[ref14] Indrawan F Nahartyo E : Analisis Penilaian Kinerja Aktivitas Tanggung Jawab Sosial dengan Metode Pengukuran Kinerja Prism dalam Perspektif Flobal Reporting Initiative (GRI) (Kasus pasa Unit Program Kemitraan dan Bina Lingkungan (PKBL) Suatu BUMN Pelabuhan). *ABIS: Accounting and Business Information Systems Journal.* 2020;7(4). 10.22146/abis.v7i4.58781

[ref15] Kelly D Holmes A Hayward R : Partnership Law. *Business Law.* 2020;4:341–366. 10.4324/9781843148043-29

[ref16] Kemenkeu: Ayo Kenali Pajak bagi pelaku UMKM. *Kemenkeu.Go.Id.* 2022. Reference Source

[ref17] Kholid M : Prinsip-Prinsip Hukum Ekonomi Syariah Dalam Undang-Undang Perbankan Syariah. *Asy-Syari’ah.* 2018;20(2):145–162. 10.15575/as.v20i2.3448

[ref18] Krisna R Yusuf M Putra E : Analysis of the halal ecosystem and halal literacy on the development of Islamic economic halal regulation. *Proceeding of International Conference on Business and Economics.* 2023;1(1):318–336.

[ref19] Mantili R : Model Of Partnership Agreement Between Medium Small Businesses (Smes) And Big Businesses In Realizing Joint Welfare. *Sociological Jurisprudence Journal.* 2020;3(1):28–33. 10.22225/scj.3.1.1514.28-33

[ref20] Michael Schaper RB : The Role of Competition Policy in Strengthening the Business Environment for MSMEs in the ASEAN Region. 2021 February; pp.1–45.

[ref21] Nikam RJ : Bespoke Crowdfunding Regulation: A Boost Up to Startups and SMEs in India. *Hasanuddin Law Review.* 2019;5(1):55–76. 10.20956/halrev.v5i1.1587

[ref22] Nugraheni N : Crowdfunding-based fiduciary warrant in providing capital loans for small and medium enterprises. *Hasanuddin Law Review.* 2020;6(3):224–231. 10.20956/halrev.v6i3.2201

[ref39] OJK: Survei Nasional Literasi dan Inklusi Keuangan 2016. *Survei Nasional Literasi Dan Inklusi Keuangan 2019.* 2016;282.

[ref23] OJK: Siaran Pers Survei Nasional Literasi Dan Inklusi Keuangan Tahun 2022. *Otoritas Jasa Keuangan.* 2022a November;10–12.

[ref24] OJK: *Press Release: The 2022 National Financial Literacy and Inclution Survey Number: Sp/82/dhms/ojk/xi/2022.* Ojk: Go.Id;2022b. Reference Source

[ref25] Keuangan OJ : Hasil Survey Nasional Literasi dan Inklusi Keuangan Tahun 2022. 2022.

[ref27] Permana A : Tantangan Dan Peluang Industri Halal Di Indonesia Dan Dunia. *Institut Teknologi Bandung.* 2019, March 18;1. Accessed May 18, 2023.

[ref28] Prabowo MS Umami YZ : the Existence of a Company in the Society and Its Legality in Indonesian Law. *Journal of Private and Commercial Law.* 2018;2(1):33–46. 10.15294/jpcl.v2i1.13962

[ref35] President Republic of Indonesia Law of the Republic of Indonesia Number 33 Year 2014 on Halal Product Assurance, Pub. L. No. Official Gazette of the Republic of Indonesia Year 2014 Number 295.2014. Reference Source

[ref29] Stiglitz JE : The Welfare State in the USSR. *State and Social Welfare, The.* 2021;205–227. 10.4324/9781315845029-20

[ref30] Sukoso W Kusnadi A Sucipto J : *Ekosistem Industri Halal.* Departemen Ekonomi dan Keuangan Syariah-Bank Indonesia Pusat Studi Halal Thoyyib-Universitas Brawijaya;2020.

[ref31] Suparji: Implementation of Intellectual Property Right to Strengthen Small and Medium-Sized Enterprise Business Capacity in Global Competition. *Academic Journal of Interdiciplinary Studies.* 2020;9(6):139–147. 10.36941/ajis.2020.v9n6r

[ref32] Suparji. : Investment climate for MSMEs towards a green economy. *International Journal of Research in Business & Social Science.* 2021;10(6):153–158. 10.20525/ijrbs.v10i6.1360

[ref46] Sauri S : Indonesia: Pemain Penting di Panggung Global Industri Halal. Febi.Uin.Antarsari. 2024, January 5. Accessed March 17, 2025. Reference Source

[ref33] Syamsu K : *Produk Halal Indonesia: Mampukah Kekuatan Domestik Memenangkan Persaingan Global?.* Pusat Kajian Sains Halal -IPB 2023, April 27. Accessed May 18, 2023. Reference Source

[ref41] Tambunan CR : Kontribusi UMKM dalam Perekonomian Indonesia. *Kantor Pelayanan Perbendaharaan Negara (KPPN) Lubuk Sikaping.* 2023, June 27. Accessed 8 January 2025.

[ref34] Tanjung KTP : Penguasaan dan Posisi Taward dalam Perjanjian Kemitraan: Sebuah Diskursus tentang “Penguasaan” dalam Perjanjian Kemitraan. *Jurnal Persaingan Usaha.* 2022;2(2):91–99. 10.55869/kppu.v2i2.56

[ref44] Tanjung Y : Peran Akademisi Dalam Meningkatkan Kualitas UMKM dan Pemasaran. *RRI-Banjarmasin.* 2024, January 21; Accessed 8 January 2025.

[ref36] Wahdiniwaty R Firmansyah D Dede SA : The Concept of Quadruple Helix Collaboration and Quintuple Helix Innovation as Solutions for Post Covid 19 Economic Recovery. *MIX: JURNAL ILMIAH MANAJEMEN.* 2022;12(3):418. 10.22441/jurnal_mix.2022.v12i3.005

[ref37] Windhyastiti I Khouroh U Aristanto E : The Role of Quadruple Helix in Supporting Sustainability of Culinary Business. *7th International Conference of Graduate School Oon Sustainability.* 2022;2009;10–14.

