# Supplementary material for: Legal aspects and government policy in increasing the role of MSMEs in the Halal ecosystem
Source: F1000Res. 2025 Mar 27;13:722. Originally published 2024 Jul 1. [Version 4] doi: 10.12688/f1000research.148322.4 (PMC11904398; doi:10.12688/f1000research.148322.4)
Supplement: Peer review history

# Reviewer report: Reviewer response for version 4

**DOI:** 10.5256/f1000research.179530.r373643

**Published:** 2025-04-04

**Authors:** Nun Maziyyah Mahsyar

**Affiliations:** 1 Sakarya University, Sakarya, Turkey

I would like to confirm that the revisions made are appropriate and in line with the required updates. Kindly it can be proceed with the approved.

Is the work clearly and accurately presented and does it cite the current literature?

Partly

If applicable, is the statistical analysis and its interpretation appropriate?

Not applicable

Are all the source data underlying the results available to ensure full reproducibility?

No source data required

Is the study design appropriate and is the work technically sound?

Partly

Are the conclusions drawn adequately supported by the results?

Yes

Are sufficient details of methods and analysis provided to allow replication by others?

No

Reviewer Expertise:

Islamic finance and green finance.

I confirm that I have read this submission and believe that I have an appropriate level of expertise to confirm that it is of an acceptable scientific standard.

# Reviewer report: Reviewer response for version 3

**DOI:** 10.5256/f1000research.176979.r369733

**Published:** 2025-03-12

**Authors:** Nun Maziyyah Mahsyar

**Affiliations:** 1 Sakarya University, Sakarya, Turkey

This paper provides valuable insights into the legal aspects and government policies supporting MSMEs in the Halal ecosystem, highlighting an important economic and regulatory development area. However, some points must be considered. 

 1. The first and second sentences of the introduction part lack logical flow. The authors first state, 'Indonesia has the potential to become a world market for halal goods and services,' but immediately follow with, 'Its failure as the largest Muslim country in the world needs attention from stakeholders.' This sudden shift is confusing because it does not explain why Indonesia failed or provide supporting data to justify this claim. A strong introduction should smoothly transition between ideas, offering explanations and evidence-based.

 2. Lack of research gap. The introduction lacks critical engagement with existing research. While it discusses MSMEs' role in the Halal economy, it does not identify what specific aspect of MSME policy, legal framework, or government support remains unexplored.

 3. The paper lacks a clear statement of novelty. While it reviews MSME challenges and government policies, it does not explain how its legal analysis differs from past studies. For example, if the paper applies the quadruple helix model to MSMEs, it should clarify that this is an existing concept and then what is new in this study and how it differs from prior research.

 4. The paper refers to OJK (Financial Services Authority), but this label is ambiguous in an international context. Instead of saying Based on OJK (Financial Services Authority) data, the authors can use Based on Indonesian Financial Services Authority (OJK) data, so that it ensures the readability for international readers.

Is the work clearly and accurately presented and does it cite the current literature?

Partly

If applicable, is the statistical analysis and its interpretation appropriate?

Not applicable

Are all the source data underlying the results available to ensure full reproducibility?

No source data required

Is the study design appropriate and is the work technically sound?

Partly

Are the conclusions drawn adequately supported by the results?

Yes

Are sufficient details of methods and analysis provided to allow replication by others?

No

Reviewer Expertise:

Islamic finance and green finance.

I confirm that I have read this submission and believe that I have an appropriate level of expertise to confirm that it is of an acceptable scientific standard, however I have significant reservations, as outlined above.

# Response

**Published:** 2025-03-13

**Authors:** Aris Machmud

**Affiliations:** Law Faculty, University Al-Azhar Indonesia, South Jakarta, Jakarta, Indonesia

Dear Nun Maziyyah Mahsyar

 Thank you for your review and recommendation, we will revise it as soon as possible.

 Best Regard

 Aris Machmud

# Response

**Published:** 2025-03-17

**Authors:** Aris Machmud

**Affiliations:** Law Faculty, University Al-Azhar Indonesia, South Jakarta, Jakarta, Indonesia

Dear, Nun Mazziyah Mahsyar,

 Thank you for your review and recommendations here are the revisions we have made:

 1. Indonesia can potentially be a leader in the global halal industry due to the supporting factor of the largest Muslim population. However, becoming a global leader in the halal industry will be lost if there is no other vital support from stakeholders, both in the form of policies and regulations related to halal certification, consumer awareness, research from academics and industry players, and community groups.

 2. Empowering MSMEs to have global competitiveness, the government issued regulations related to guaranteeing gratuitous halal products besides that, the government applies low taxation norms where the tax rate subsidy is zero point five percent for turnover up to 4.8 billion rupiahs as well as a loan interest subsidy of five percent and provides support for the digitization of the MSME market, as well as increasing business cooperation between large companies and MSMEs on a reciprocal basis that is accountable for realizing an integrated halal product supply chain (Supian Sauri, 2024).To improve the competitiveness of MSMEs in addition to those mentioned above, the government assists in adequate training and promotion (Hidayat et al., 2025).

 3. Kelemahan dari penelitian Suparji adalah cenderung lebih menekankan pada reformasi hukum yang adaptif terhadap segala perubahan lingkungan bisnis untuk mendukung ekosistem ekonomi hijau dan pengembangan bisnis UMKM melalui fasilitas dan kebijakan pemerintah. Globalisasi dan pasar bebas membawa persaingan yang ketat, dimana hanya produk yang memiliki keunggulan kompetitif yang dapat bertahan dalam dunia bisnis, termasuk UMKM. Kelemahan dari penelitian Asep Firmansyah menyatakan bahwa UMKM lebih mengutamakan pangsa pasar dalam negeri. Dalam penelitian Hartono, UMKM dapat menjadi pemain utama dalam ekosistem halal dunia melalui kolaborasi dan pemanfaatan media digital dalam sistem keuangan dan akses pasar. Artikel Benuf & Azhar dan Ali mengatakan bahwa UMKM dapat berkembang dengan transformasi ekosistem produk halal dan orientasi baru dalam pembentukan pasar industri halal. Penelitian Nugraheni menekankan bahwa UMKM masih kalah bersaing karena kurangnya akses pembiayaan dan sertifikasi halal.

 Berdasarkan state of the art di atas, penulis menyatakan bahwa terdapat perbedaan pada objek penelitian dan ruang lingkup penelitian terkait UMKM. Kebaruan dari penelitian ini adalah meningkatkan peran UMKM dalam mendukung Indonesia menjadi pemimpin dan menguasai pangsa pasar industri produk dan jasa halal global.

 4. Otoritas Jasa Keuangan (OJK)

# Response

**Published:** 2025-03-21

**Authors:** Aris Machmud

**Affiliations:** Law Faculty, University Al-Azhar Indonesia, South Jakarta, Jakarta, Indonesia

Dear, Nun Mazziyah Mahsyar,

 Thank you for your review and recommendations here are the revisions we have made:

**1. The first and second sentences of the introduction part lack logical flow. The authors first state, 'Indonesia has the potential to become a world market for halal goods and services,' but immediately follow with, 'Its failure as the largest Muslim country in the world needs attention from stakeholders.' This sudden shift is confusing because it does not explain why Indonesia failed or provide supporting data to justify this claim. A strong introduction should smoothly transition between ideas, offering explanations and evidence-based.**

 Response: Indonesia can potentially be a leader in the global halal industry due to the supporting factor of the largest Muslim population. However, becoming a global leader in the halal industry will be lost if there is no other vital support from stakeholders, both in the form of policies and regulations related to halal certification, consumer awareness, research from academics and industry players, and community groups.

**2. Lack of research gap. The introduction lacks critical engagement with existing research. While it discusses MSMEs' role in the Halal economy, it does not identify what specific aspect of MSME policy, legal framework, or government support remains unexplored.**

 Response: Empowering MSMEs to have global competitiveness, the government issued regulations related to guaranteeing gratuitous halal products besides that, the government applies low taxation norms where the tax rate subsidy is zero point five percent for turnover up to 4.8 billion rupiahs as well as a loan interest subsidy of five percent and provides support for the digitization of the MSME market, as well as increasing business cooperation between large companies and MSMEs on a reciprocal basis that is accountable for realizing an integrated halal product supply chain (Supian Sauri, 2024).To improve the competitiveness of MSMEs in addition to those mentioned above, the government assists in adequate training and promotion (Hidayat et al., 2025).

**3. The paper lacks a clear statement of novelty. While it reviews MSME challenges and government policies, it does not explain how its legal analysis differs from past studies. For example, if the paper applies the quadruple helix model to MSMEs, it should clarify that this is an existing concept and then what is new in this study and how it differs from prior research.**

 Response: The weakness of Suparji's research is that it tends to emphasize legal reforms that are adaptive to any changes in the business environment to support the green economy ecosystem and MSME business development through government facilities and policies. Globalization and free markets bring intense competition, where only products that have a competitive advantage can survive in the business world, including MSMEs. The weakness of Asep Firmansyah's research states that MSMEs prioritize domestic market share. In Hartono's research, MSMEs can become major players in the world's halal ecosystem through collaboration and utilization of digital media in the financial system and market access. Benuf & Azhar and Ali's articles say that MSMEs can develop with the transformation of the halal product ecosystem and a new orientation in the formation of the halal industry market. Nugraheni's research emphasizes that MSMEs are still unable to compete due to a lack of access to financing and halal certification.

 Based on the state of the art above, the author states that there are differences in the object of research and the scope of research related to MSMEs. The novelty of this research is to increase the role of MSMEs in supporting Indonesia to become a leader and control the market share of the global halal products and services industry.

**4. The paper refers to OJK (Financial Services Authority), but this label is ambiguous in an international context. Instead of saying Based on OJK (Financial Services Authority) data, the authors can use Based on Indonesian Financial Services Authority (OJK) data, so that it ensures the readability for international readers.**

 Response: Financial Services Authority (OJK)

# Reviewer report: Reviewer response for version 3

**DOI:** 10.5256/f1000research.176979.r361698

**Published:** 2025-02-06

**Authors:** Muhammad Anwar Fathoni

**Affiliations:** 1 Islamic Economics, Universitas Pembangunan Nasional Veteran Jakarta, Jakarta, Indonesia, Indonesia

I appreciate the authors’ efforts in revising the manuscript based on the feedback provided. The necessary improvements have been made, and the revisions effectively address the concerns raised in the previous review. The clarity, coherence, and overall quality of the paper have significantly improved. I have no further major concerns regarding the manuscript.

Is the work clearly and accurately presented and does it cite the current literature?

Yes

If applicable, is the statistical analysis and its interpretation appropriate?

Yes

Are all the source data underlying the results available to ensure full reproducibility?

Yes

Is the study design appropriate and is the work technically sound?

Partly

Are the conclusions drawn adequately supported by the results?

Partly

Are sufficient details of methods and analysis provided to allow replication by others?

Yes

Reviewer Expertise:

Islamic economics, Islamic consumer behavior, halal industry

I confirm that I have read this submission and believe that I have an appropriate level of expertise to confirm that it is of an acceptable scientific standard.

# Response

**Published:** 2025-02-06

**Authors:** Aris Machmud

**Affiliations:** Law Faculty, University Al-Azhar Indonesia, South Jakarta, Jakarta, Indonesia

Dear Muhammad Anwar Fathoni,

 Thank you for the suggestions for improvement and the approval given to our version 3 article.

 Best regards, 

 Aris Machmud

# Reviewer report: Reviewer response for version 2

**DOI:** 10.5256/f1000research.176056.r354842

**Published:** 2025-01-08

**Authors:** Mohamed Syazwan Ab Talib

**Affiliations:** 1 UBD School of Business and Economics, Universiti Brunei Darussalam, Gadong, Brunei

The paper provides insights into the role of MSMEs in Indonesia’s halal ecosystem and growth potential. However, it has some notable weaknesses:

 1. **Methodological Limitations**: The study relies exclusively on normative juridical research, which may limit its ability to address practical implications and provide empirical evidence. The absence of primary data (e.g., surveys and interviews) weakens the robustness of the findings and restricts its relevance for stakeholders.

 2. **Ambiguity in Key Arguments**: While the quadruple helix model is emphasized as a solution, the discussion lacks specificity in detailing how each stakeholder—government, academia, industry, and community—can practically collaborate. The paper does not critically assess the feasibility of implementing this model in Indonesia’s socio-economic context.

 3. **Overgeneralization**: The conclusions drawn about the potential of MSMEs are overly optimistic without addressing critical challenges such as inadequate access to financing, the lack of skilled personnel, and the limited impact of existing policies. The absence of a detailed SWOT analysis undermines the paper’s strategic contributions.

 4. **Insufficient Novelty**: Many ideas discussed, such as the importance of financial inclusion and halal certification, are well-established in existing literature. The paper lacks innovative or groundbreaking perspectives to make it stand out in this field.

 5. **Data Gaps**: While the paper mentions statistics (e.g., MSMEs' contributions to GDP), it does not substantiate these with comprehensive or recent data. Additionally, empirical validation or case studies do not support the claim that the quadruple helix system can enhance MSME competitiveness.

Is the work clearly and accurately presented and does it cite the current literature?

Partly

If applicable, is the statistical analysis and its interpretation appropriate?

Not applicable

Are all the source data underlying the results available to ensure full reproducibility?

No source data required

Is the study design appropriate and is the work technically sound?

Partly

Are the conclusions drawn adequately supported by the results?

Yes

Are sufficient details of methods and analysis provided to allow replication by others?

No

Reviewer Expertise:

Halal business management

I confirm that I have read this submission and believe that I have an appropriate level of expertise to confirm that it is of an acceptable scientific standard, however I have significant reservations, as outlined above.

# Response

**Published:** 2025-01-13

**Authors:** Aris Machmud

**Affiliations:** Law Faculty, University Al-Azhar Indonesia, South Jakarta, Jakarta, Indonesia

Dear Mohamed Syazwan Ab Talib

 Thank you for the review and recommendations. We will then send a revised article. The responses related to the review results:

 1. In legal research.

 The method often used is normative juridical with a statutory regulatory approach. However, it can used nomos and empiricism to become empirical normative juridical. In our research, the methodology used is normative juridical, the use of a multidisciplinary or interdisciplinary approach can be considered in further research.

 2. The role of inter-helix in the development of MSMEs

 a. The role of the government in government policies and regulations in supporting MSMEs is a study of whether the norm is in favor of the development of MSMEs, such as tax regulations where the government provides relief in the form of a one percent tax for MSMEs with a maximum turnover of 4.8 billion per year is enough to help MSMEs. Then President Prabowo's program to relax MSME debt and the regulations of the Ministry of Cooperatives and MSMEs encouraged the development of MSMEs in addition to government policies to collaborate between relevant institutions.

 b. Academic helix. 

 Its role in developing teaching, research, and community services can provide input to MSMEs to increase their capacity and capabilities in terms of marketing and management innovation so that they can increase their business level so that they can be bankable and gain access to financing from formal financial institutions that can provide capital facilities - because one of the obstacles to unbankable is the limitations in management, one of which is marketing and financial management including the preparation of MSME financial reports and assistance with financial and tax audits.

 c. The helix of the industrial world is through mutually beneficial assistance and collaboration - in terms of accommodating and marketing MSME products.

 d. The role of the community or society.

 The community in the halal product ecosystem produced by MSMEs is making goods produced by MSMEs a choice in meeting their life needs because their role is very crucial in supporting the development of MSMEs in Indonesia. That the role of each helix will be in vain if there is no close relationship and mutual support together.

 3. Regarding overgeneralization.

 In the case of MSMEs, there are several constraints in terms of capacity and capability so that the involvement of the four helixes (Quadruple helix) is a solution and not a fantasy and relying on pessimistic conditions that occur, many efforts have been made by various stakeholders to bridge the gap in MSMEs.

 4. The novelty in this study is that the quadruple helix system can be a solution to increasing the capacity and capability of MSMEs in the global halal industry.

 5. Data gap. We realize that normative legal research does not recognize data but relies more on primary and secondary legal sources to answer existing problems, but in social research, data sources are primary

 Best Regard

 Aris Machmud

# Reviewer report: Reviewer response for version 2

**DOI:** 10.5256/f1000research.176056.r354847

**Published:** 2025-01-08

**Authors:** Muhammad Anwar Fathoni

**Affiliations:** 1 Islamic Economics, Universitas Pembangunan Nasional Veteran Jakarta, Jakarta, Indonesia, Indonesia

**Comments and General Review**

1. **Clarity and Focus of Writing:** The article covers essential aspects of MSMEs' roles in the halal ecosystem, including legal, governmental policy, and the Quadruple Helix system. However, some sections feel repetitive, particularly regarding challenges and potential MSMEs face in the halal ecosystem. The structure could be improved for better flow, especially in the results and discussion sections, which tend to merge without clear boundaries.
2. **Methodology:** The normative juridical research approach is appropriate for exploring regulations and policies. However, there is room to elaborate on how the data was analyzed within the research context. The heavy reliance on secondary sources leaves the study lacking primary data, such as interviews or surveys, which could strengthen the arguments.
3. **Results and Discussion:** The findings clearly highlight MSME challenges, such as access to capital, financial literacy, and halal certification. However, the discussion on solutions, such as the implementation of the Quadruple Helix, lacks depth and practical application. Further elaboration is needed on how the government, academics, industry, and society can collaborate in practical terms to improve MSME competitiveness.
4. **References:** The references are comprehensive and relevant. However, some parts rely on secondary sources that are general and not specifically tailored to Indonesia's context. The article could benefit from including more recent empirical studies to support the arguments.

**Technical Comments**

**Language and Grammar:** The language used is formal and academic, but some sentences are overly long and complex, making it challenging for readers to grasp the main points. There is repetitive use of terms like "halal ecosystem," which could be simplified or diversified.

**Suggestions for Improvement**

1. **Sharpen Focus:** Reduce repetitive content in sections like the background and discussion. Focus on key points that support the research objectives.
2. **Detail Solutions:** Provide more in-depth elaboration on the implementation of the Quadruple Helix system and how each element can concretely contribute to MSME development in the halal ecosystem.
3. **Conclusion:** Strengthen the conclusion with more practical recommendations for stakeholders, especially the government and MSME actors.

Is the work clearly and accurately presented and does it cite the current literature?

Yes

If applicable, is the statistical analysis and its interpretation appropriate?

Yes

Are all the source data underlying the results available to ensure full reproducibility?

Yes

Is the study design appropriate and is the work technically sound?

Partly

Are the conclusions drawn adequately supported by the results?

Partly

Are sufficient details of methods and analysis provided to allow replication by others?

Yes

Reviewer Expertise:

Islamic economics, Islamic consumer behavior, halal industry

I confirm that I have read this submission and believe that I have an appropriate level of expertise to confirm that it is of an acceptable scientific standard, however I have significant reservations, as outlined above.

# Response

**Published:** 2025-01-13

**Authors:** Aris Machmud

**Affiliations:** Law Faculty, University Al-Azhar Indonesia, South Jakarta, Jakarta, Indonesia

Dear Muhammad Anwar Fathoni

 Thank you for the review and recommendations. Here are the responses to the review results:

 1. Clarity and Focus of Research: In this study, we have improved it by dividing it into sub-themes, and we have improved the suggestions and improvements in the revised article.

 2. Methodology: in normative legal research, there is no data, except for the method used is empirical juridical, which uses two approaches quantitatively and qualitatively. However, the existing data is only a tool for answering problems. The normative juridical method is different from other social research. The availability of data is the main source of social research. Normative juridical research emphasizes the approach of statutory regulations with primary and secondary legal sources obtained through literature studies.

 3. Results and discussion: the involvement of each helix - government, academics, society, and the industrial world - has been explained in the revised article.

 4. References: relevant and comprehensive references, the use of a multidisciplinary or interdisciplinary approach can be considered in further research.

 Best Regard

 Aris Machmud
